# Exploration of microbiome diversity of stacked fermented grains by flow cytometry and cell sorting

**DOI:** 10.3389/fmicb.2023.1160552

**Published:** 2023-03-27

**Authors:** Ziyang Zhang, Yanwei Wei, Zehao Peng, Peng Du, Xinyong Du, Guoying Zuo, Chaoqing Wang, Piwu Li, Junqing Wang, Ruiming Wang

**Affiliations:** ^1^State Key Laboratory of Biobased Material and Green Papermaking, Qilu University of Technology, Jinan, Shandong, China; ^2^Department of Biological Engineering, Qilu University of Technology, Jinan, Shandong, China; ^3^Gubeichun Group Co., Ltd., Jinan, Shandong, China

**Keywords:** flow cytometry, cell sorting, high-throughput sequencing, microbiome diversity, stacked fermented grains

## Abstract

Sauce-flavor baijiu is one of the twelve flavor types of Chinese distilled fermented product. Microbial composition plays a key role in the stacked fermentation of Baijiu, which uses grains as raw materials and produces flavor compounds, however, the active microbial community and its relationship remain unclear. Here, we investigated the total and active microbial communities of stacked fermented grains of sauce-flavored Baijiu using flow cytometry and high-throughput sequencing technology, respectively. By using traditional high-throughput sequencing technology, a total of 24 bacterial and 14 fungal genera were identified as the core microbiota, the core bacteria were *Lactobacillus* (0.08–39.05%), *Acetobacter* (0.25–81.92%), *Weissella* (0.03–29.61%), etc. The core fungi were *Issatchenkia* (23.11–98.21%), *Monascus* (0.02–26.36%), *Pichia* (0.33–37.56%), etc. In contrast, using flow cytometry combined with high-throughput sequencing, the active dominant bacterial genera after cell sorting were found to be *Herbaspirillum*, *Chitinophaga*, *Ralstonia*, *Phenylobacterium*, *Mucilaginibacter*, and *Bradyrhizobium*, etc., whereas the active dominant fungal genera detected were *Aspergillus*, *Pichia*, *Exophiala*, *Candelabrochaete*, *Italiomyces*, and *Papiliotrema*, etc. These results indicate that although the abundance of *Acetobacter*, *Monascus*, and *Issatchenkia* was high after stacked fermentation, they may have little biological activity. Flow cytometry and cell sorting techniques have been used in the study of beer and wine, but exploring the microbiome in such a complex environment as Chinese baijiu has not been reported. The results also reveal that flow cytometry and cell sorting are convenient methods for rapidly monitoring complex microbial flora and can assist in exploring complex environmental samples.

## Introduction

Microbiome research that focuses on exploring the structure and function of microbial flora has gained considerable attention over the past 20 years. Considerable effort has been devoted to exploring the structure and function of flora. Microorganisms in different environments have been enriched and domesticated over time to form specific microbial communities and play specific roles. For example, intestinal microbial flora is involved in the growth and development of the body and in maintaining intestinal homeostasis (Martinez-Martinez et al., 2022); Microbes adapted to the soil ecosystem play crucial roles in soil structure formation and soil and crop health by promoting the release of nutrients and suppressing soil-borne pathogens (Liu et al., 2023); the microbial community in fermented foods breaks down raw materials and imparts a variety of flavors (Wu et al., 2021). Microorganisms represent a large reservoir of genetic diversity; even the smallest natural microbial communities contain thousands of complex microorganisms. Although culture-based methods have been used widely to study complex microbial communities, they are limited to their biased detection as the vast majority of these microorganisms cannot be studied under laboratory culture conditions (Koch et al., 2013). Nevertheless, the advent of molecular biology techniques, such as real-time polymerase chain reaction PCR (Fibi et al., 2016), PCR-denaturing gradient gel electrophoresis (Pindi et al., 2019), high-throughput sequencing (Syromyatnikov et al., 2022) and metagenomics (Siles et al., 2022), has helped overcome the limitations of traditional isolation and culture techniques.

Flow cytometry is a technique for the rapid analysis of the physical and chemical properties of cells, using light scattering and fluorescence measurements (Muller and Nebe-von-Caron, 2010; Hatzenpichler et al., 2020; Rubbens and Props, 2021), it has been widely used in medicine (Putheti et al., 2022; Sur et al., 2022). In recent years, flow cytometry has also been applied to characterize multicellular fungi and complex microbial communities (Bleichrodt and Read, 2019; Rubbens et al., 2021). In contrast to the current gold standard high-throughput sequencing technology for microbial community detection, flow cytometry detects single cells, and enables the real-time monitoring of microbial communities by rapidly collecting cellular data and storing them in a special file format, however, studies exploring the applicability of flow cytometry in complex matrices are limited (Rubbens et al., 2021).

Baijiu is a traditional Chinese famous distilled fermented product with unique flavors and several health benefits. It is prepared by brewing cereals as raw materials in the presence of a fermenting agent (wine malt). Baijiu is processed using unique fermentation technologies in an open unsterilized environment that utilizing several microorganisms that play key roles in imparting the unique flavors of Baijiu (Niu et al., 2022). Stacked fermentation is a unique process for obtaining sauce-flavor Baijiu. The presoaked, steamed, and cooled raw materials are mixed with Daqu (fermentation starter) and the mixture is then stacked on piles in an open environment and allowed to ferment naturally. The stacking enhances microbial activities, leading to heat generation and further enrichment of microorganisms that play crucial roles in subsequent alcoholic fermentation steps in the cellar, producing saucy white wine flavor, and precursor substances, etc., (Huang et al., 2017). Thus, stacking fermentation involves a plethora of microorganisms forming complex microbial communities. However, the functional structure of microorganisms and the mechanism of stacked fermentation are poorly understood. Therefore, in this study, we aimed to investigate the dynamic changes in the microbial community during the stacked fermentation of Baijiu using flow cytometry and high throughput sequencing techniques. This study will provide a rapid and convenient real-time monitoring technique to profile microorganisms during long-term fermentation. Our findings can assist in brewing quality control and technology improvement, as well as provide a basis for the flow cytometry analysis of microorganisms in more complex environments.

## Materials and methods

### Experimental materials

Stacked samples of sauce-flavored Baijiu were obtained from first to third rounds of stacked fermented grains from the Gubeichun Group Co., Ltd., in Shandong, China. The samples were named DJ1, DJ2, and DJ3 on the first day of the stack, and DJ1RJ, DJ2RJ, and DJ3RJ on the last day of the stack. The samples were sealed in sterile bags and sent to the laboratory for testing as soon as possible.

### Culture and handling of model microorganisms

The model microorganisms, including *Escherichia coli*, *Corynebacterium glutamicum*, *Bacillus subtilis*, *Clostridium celerecrescens*, *Pichia pastoris*, *Saccharomyces cerevisiae*, and *Aureobasidium pullulans* were incubated in the respective media for 12 h. The model microorganisms were cultured for 12 h and filtered through a 300-mesh filter membrane, and 1 mL of the suspension was used for the flow-through analysis. The model microorganisms were obtained from strains available in our laboratory.

A mixture of 1.5 μL SYTO9 (Invitrogen, United States) with 1.5 μL PI (Invitrogen, United States) dye was added to 1 mL of the sample suspension, mixed well, and incubated for 15 min in the dark. The above operation was used for the microbial activity analysis.

### Extraction method and optimization of microorganisms from stacked fermented grains

The stacked fermented grains (5 g) and 0.85% sterilized sodium chloride solution (30 mL) were added to a 50 mL sterilized centrifuge tube and vortexed with three glass beads with a diameter of 5–7 mm (Hu et al., 2021). The solutions were collected after 0, 5, 10, 15, 20, and 25 min of shaking and filtered using a 300-mesh filter membrane. Subsequently, the filtered suspension (1 mL) was set aside for the flow-through analysis. The samples were prepared to count and assess active microorganisms by adding 20 μL of mixed standard microspheres (Meilun Bio, China) to 977 μL of the stacked fermented grain sample suspension. Counting microspheres of known size and fluorescence were captured in an FSC (correlates with cell size)-FITC(green fluorescent) two-parameter map by manually adding a rigorous selection of microspheres of known size and fluorescence to the sample. Next, 1.5 μL of SYTO9 and 1.5 μL of PI dye were added to this mixture, which was incubated for 15 min in the dark.

### Flow cytometry analysis

The flow cytometer used for the experiments used 0.85% superior pure sodium chloride as the sheath solution, and the optical and liquid flow systems of the hybrid fluorescent microspheres (Beckman Coulter, Brea, CA, United States) used for the flow cytometer were quality controlled for the optical path. The prepared samples were then subjected to flow cytometric analysis using a Moflo XDP ultrafast flow cytometer with a three-color laser The flow cytometer used for the experiments used 0.85% superior pure sodium chloride as the sheath solution, and the optical and liquid flow systems of the hybrid fluorescent microspheres (Beckman Coulter, Brea, CA, United States) used for the flow cytometer were quality controlled for the optical path. The prepared samples were then subjected to flow cytometric analysis using a Moflo XDP ultrafast flow cytometer with a three-color laser (Beckman Coulter, Brea, CA, United States). The maximum excitation wavelengths of both SYTO9 and PI are approximately 488 nm; therefore, a 488 nm laser was used to excite both dyes efficiently. The maximum emission wavelength of SYTO9 was approximately 500 nm, and that of PI was approximately 635 nm. The FL1 and FL3 channels were chosen to collect the emission wavelengths. The sheath-to-sample pressure difference was adjusted to ensure that the number of targets detected per second was approximately 1,000. The FSC and FITC thresholds were set at 0.1 and 0.05, respectively, and the reaction was stopped when 50,000 cells were collected.

The collected data were analyzed and processed using Kaluza, a dedicated data analysis software for the MOFLO XDP flow cytometer.

### DNA extraction, amplification, and sequencing

DNA extraction from stacked fermented grains was carried out using the E.Z.N.A™ Mag-Bind Soil DNA Kit (OMEGA, United States), following the kit’s instructions. Briefly, 30 mL of 0.1 mol/L phosphate buffered saline (PBS)buffer was added to 5 g of stacked fermented grains, this solution was mixed with three glass beads, shaken for 5 min, and centrifuged at 300 r/min for 5 min to obtain the supernatant. The precipitate was washed three times with PBS and centrifuged to collect the supernatant. Subsequently, the supernatants collected at both steps were pooled and centrifuged at 9,000 r/min. After discarding the supernatant, the precipitate was collected, washed three times with PBS, centrifuged at 9,000 r/min for 3 min, and resuspended in 2 mL of PBS.

For microbial community analysis, the DNA extracted from stacked fermented grains was used to amplify the 16S r RNA V3-V4 region of bacteria using primers 341F (5′-CCTA CGGGNGGCWGCAG-3′) and 805R (5′-GACTACHVGGGTATC TAATCC-3′) and the ITS1-ITS2 region of fungal using the primers ITS1F (5′-CTTGGTCATTTAGAGGAAGTAA-3′) and ITS2R (5′-GCTGCGTTCTTCATCGATGC-3′). The PCR system was as follows: 15 μL 2 × Hieff Robust PCR Master Mix, 1 μL 10 μM sense primer, 1 μL 10 μM antisense primer, 10 ng genomic DNA, and supplemented to 30 μL with ddH2O. The PCR condition was as follows: 94°C for 3 min, 5 cycles (94°C for 30 s, 45°C for 20 s, 65°C for 30 s), 20 cycles (94°C for 20 s, 55°C for 20 s, 72°C for 30 s), and 72°C for 5 min. The PCR products were subjected to high-throughput sequencing using the Illumina MiSeq sequencing platform by Sangon Biotech (China). Gel images are shown in Supplementary Figures 1–4.

## Results

### Flow cytometry analysis of model microorganisms

**FIGURE 1:**
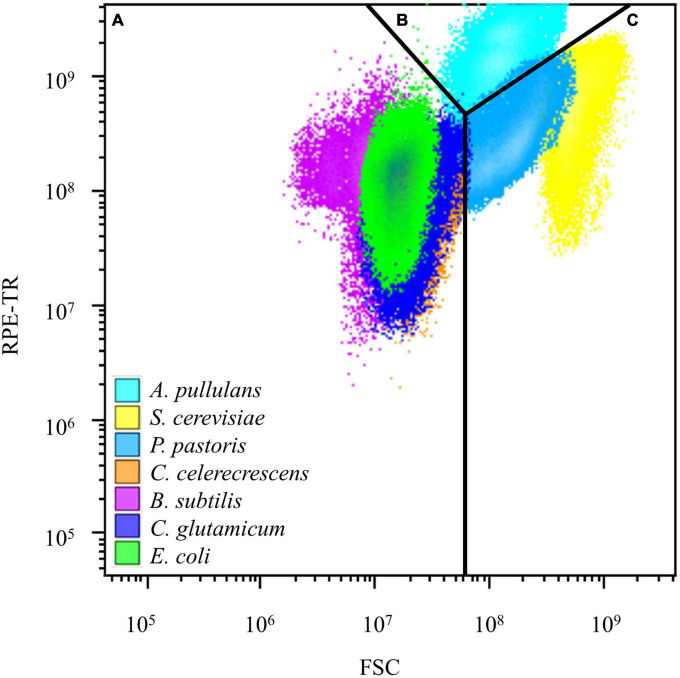
Results of flow cytometry analysis of model microorganisms. **(A)** Metabolically active bacteria distributed in the region after flow cytometry analysis. **(B)** Metabolically active mold in the region after flow cytometry analysis. **(C)** Metabolically active yeast distributed in the region after flow cytometry analysis.

In this study, the two-parameter fluorescence channel analysis revealed that bacteria such as *E. coli* and *C. glutamicum* exhibited relative FSC below 10^8^ and RPE-TR (red fluorescence) fluorescence values below 10^9^. *S. cerevisiae* and *P. pastoris* staining showed FSC values above 10^8^, and *A. pullulans* showed RPE-TR values above 10^9^ in the two-parameter plots. These results showed that bacteria, yeast, and mold formed subgroups in the FSC-RPE-TR parameter map after double staining with nucleic acid dyes (Figure 1).

### Flow cytometry analysis of stacked fermented grains

**FIGURE 2:**
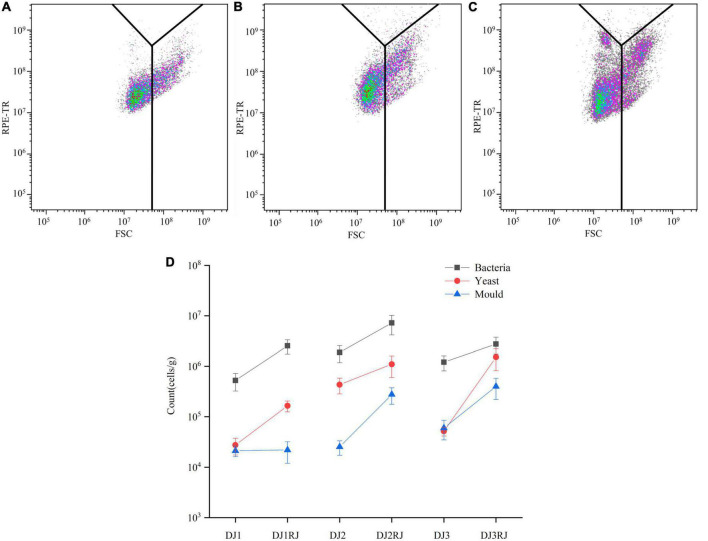
Microbial changes during rounds 1–3 of stacking by flow cytometry analysis. **(A)** Flow cytometric analysis of the first round of stacked grain activity staining. **(B)** Flow cytometric analysis of the second round of stacked grain activity staining. **(C)** Flow cytometric analysis of the third round of stacked grain activity staining. **(D)** Changes in the number of microorganisms in 1–3 rounds of stacked fermented grains.

Nucleic acid-dye staining of stacked fermented grains revealed an increase in FITC fluorescence intensity in active cells with intact cell membranes, whereas no significant change in FITC fluorescence intensity was observed in dead cells; thus, the samples of stacked fermented grains could be differentiated for activity. The active cells were further analyzed using Kaluza software. Overlaying the FSC-RPE-TR dual parameter data of fermented grain samples with those of the model microorganisms in the two-parameter plots revealed that the fermented grain samples were scattered in various bacterial, yeast, and mold regions (Figures 2A–C).

We manually added 20,000 counted microspheres to the samples, calculated the proportion of counted microspheres in the collected events, and further calculated the trend of microorganisms in the fermented grain samples, as shown in Figure 2D. As the stacking progressed, the total number of microorganisms increased compared to that at the beginning of the stacking phase, with the percentage of increase varying between the stacking rounds, especially for yeast, which increased by 10–100 times. However, the number of bacteria remained the highest throughout the stacking process.

### Optimization results for the microbial extraction of baijiu stack samples

**FIGURE 3:**
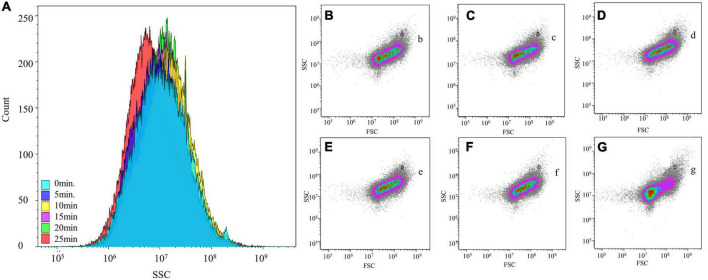
Flow cytometry analysis of different extraction times on stacked fermented grains. **(A)** Effect of different shaking times on the internal density of cell. **(B–G)** The effect of shaking time of 0, 5, 10, 15, 20, and 25 min on the microbial morphology of stacked fermented grains by flow cytometry. (b–g) Counted microspheres were detected at different shaking times.

By extracting microorganisms at different vortex shaking times, the maximum number of microorganisms was extracted after 20 min of vortex shaking, and the most concentrated SSC (correlates with cell density) parameters were obtained, however, after 25 min of shaking, a change in SSC values was observed in the SSC count plot Figure 3. This result indicated that after a long period of shaking, the morphology of some cells was changed by the repeated impact of the glass beads. Moreover, the addition of counting microspheres showed that their proportion was minimal at 20 min of shaking and constant at 25 min. Based on these results, we inferred that the extraction of microorganisms after 20 min of shaking was more satisfactory than that at other time points.

### Sequencing results

The coverage indices for all tested samples were greater than 0.99, indicating that most species were detected, providing a valid response to the microorganisms contained in the samples and a reasonable depth of sequencing. The alpha diversity indices of the stacked samples are listed in Table 1. Alpha diversity analysis identified the highest number of bacterial OTUs in the DJ1 sample and the lowest in the DJ3RJ sample. The higher the Shannon index, the higher the diversity of the sample microbial community; the higher the Simpson index, the lower the diversity of the microbial community in the sample. The results showed that sample DJ2 had the highest diversity of bacterial communities among the samples, whereas DJ3RJ had the lowest. The highest number of fungal OTUs was detected in DJ3 and the lowest in DJ3RJ, with the highest diversity of fungal species in DJ2 and the lowest in DJ2RJ.

**TABLE 1:** Alpha diversity of bacteria and fungi from stacked fermented grains.

Type	Sample	OTUs	Shannon	Chao	Ace	Simpson	Coverage
Bacteria	DJ1	181.00	3.06	193.75	190.32	0.11	0.99
Bacteria	DJ1RJ	164.00	2.44	184.65	180.87	0.17	0.99
Bacteria	DJ2	174.00	3.43	184.00	182.50	0.06	0.99
Bacteria	DJ2RJ	148.00	2.65	149.88	150.38	0.13	0.99
Bacteria	DJ3	140.00	2.42	148.27	148.61	0.16	0.99
Bacteria	DJ3RJ	118.00	0.94	126.00	130.42	0.67	0.99
Fungi	DJ1	64.00	1.47	77.33	99.19	0.36	0.99
Fungi	DJ1RJ	59.00	1.87	80.00	88.01	0.23	0.99
Fungi	DJ2	123.00	1.36	140.14	132.22	0.50	0.99
Fungi	DJ2RJ	39.00	0.14	56.00	87.05	0.96	0.99
Fungi	DJ3	215.00	1.30	216.56	217.34	0.54	0.99
Fungi	DJ3RJ	21.00	1.27	26.00	26.30	0.31	0.99

Except for a slight increase in the Shannon indices of the fungi at the end of the first round, at the end of each stack, the aroma and intensity indices of bacteria and fungi decreased, and community diversity declined. This indicates that many microorganisms that are intolerant to high temperatures could have died during the stacking process, resulting in a decrease in community diversity, thereby creating an enrichment of microorganisms required for cellar fermentation. The OTU and Shannon indices at the end of stacking varied from round to round, indicating differences in microorganism diversity between the rounds of brewing and fermentation in the same batch.

### Bacterial microbial composition of stacked fermented grains

In this study, those with relative abundance greater than 1% were considered dominant species, and those with less than 1% were classified as other.

**FIGURE 4:**
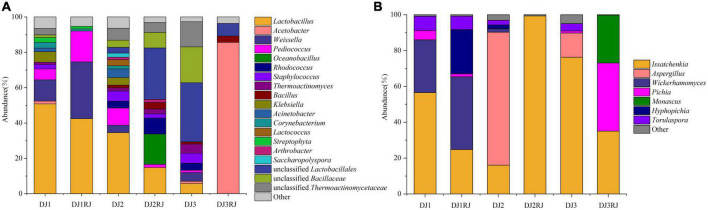
Microbial abundance in 1–3 rounds of stacked fermented grains based on high-throughput sequencing data. **(A)** Bacterial abundance map for stacking rounds 1–3 of stacked fermented grains. **(B)** Fungal abundance map for stacking rounds 1–3 of stacked fermented grains.

In the six stacked samples, 117 bacterial genera were detected, of which 24 were dominant (greater than 1% abundance in at least one sample). The main bacterial phyla were *Firmicutes* and *Proteobacteria*, which accounted for 85.65–98.28% of the total bacterial abundance in the six samples. *Lactobacillus, Acetobacter, Weissella, Pediococcus, Bacillaceae*, and *Thermoactinomycetacea* accounted for 58.61–89.28% of the total bacterial content and were considered the dominant core genera. Data on the dynamics of the specific genera are shown in Figure 4A. Of these, *Lactobacillus* was the dominant genus until the end of the third round of stacking, and its proportion decreased gradually with an increased stacking round (47.66–5.42%). *Weissella* was more abundant during the first round of accumulation, and at the beginning of the second and third rounds, *Pediococcus* was effectively enriched at the end of the first round. *Oceanobacillus* and *Rhodococcus* became dominant at the end of the second round. *Acetobacter* became the dominant bacterial genus (81.92%) in the three sub-accumulation phases, whereas *Komagataeibacter* was elevated and only detected in trace amounts in the rest of the accretion phase, being the dominant genus specific to the three sub-accumulation phases.

### Composition of fungal microorganisms of stacked fermented grains

The dominant fungal phylum in the stacked fermented grain samples was *Ascomycota* which accounted for 95.69–99.99% of the fungal abundance in each sample. A total of 142 fungal genera were detected, of which 14 were dominant, *Issatchenkia, Aspergillus, Wickerhamomyces, Pichia*, and *Monascus* accounted for 63.11–99.16% of the fungal abundance in each sample and were the core fungal genera in rounds 1–3 of the stacking process. *Issatchenkia* was the dominant fungal genus in all three rounds (15.23–98.21%), whereas *Wickerhamomyces* and *Hyphopichia* were enriched in one round of accumulation and became the absolute dominant strains before cellar fermentation. The abundance of *Issatchenkia* increased in the second round of stacking and became the dominant genus before cellular fermentation, whereas that of *Aspergillus* decreased significantly during the stacking process. Three rounds of accumulation were enriched with a large number of *Pichia* and *Monascus* (Figure 4B).

### Cytometric sorting of microbes

**FIGURE 5:**
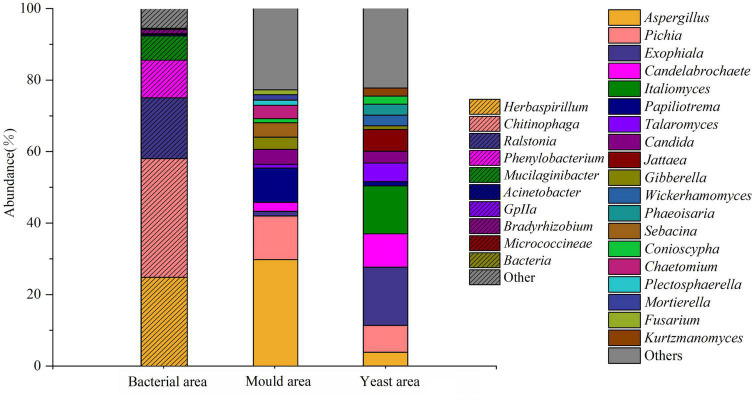
Map of microbial abundance in different areas after sorting samples at the end of the third round of stacking.

By developing a method to detect live bacteria in stacked fermented grains using flow cytometry, we could cytometrically sort and sequence delineated regions of bacteria, yeast, and fungi. The DNA extraction results showed that the fungal genomic bands in the bacterial region were extremely dark, indicating that very few fungi were sorted from this region. In contrast, the bands in the mold and yeast regions were normal. In total, 263 bacterial and 201 fungal genera were detected *via* sequencing. The main active bacteria were *Herbaspirillum* (24.93%), *Chitinophaga* (33.23%), *Ralstonia* (17.01%), *Phenylobacterium* (10.52%), *Mucilaginibacter* (6.79%), and *Bradyrhizobium* (1.21%), *Aspergillus* (30.09%) was the dominant fungus in the mold region, whereas *Gibberella* (3.40%), *Chaetomium* (3.73%), *Mortierella* (1.49%), *Fusarium* (1.36%), and many other mold species were enriched in this area. As shown in Figure 5, the dominant yeast genera present in the yeast region were *Italiomyces* (13.42%), *Pichia* (7.55%), *Candida* (3.31%), *Wickerhamomyces* (3.98%), and *Kurtzmanomyces* (2.27%). Direct sequencing of samples without cytometric sorting identified the microorganisms present in the sample. In contrast, staining and cytometric sorting identified the active microorganisms in their respective stages. The results of these two methods showed considerable differences.

## Discussion

Microorganisms in the fermentation process exist in both dead and living forms, and only active microorganisms can metabolize substances that affect the surrounding environment (Cimini et al., 2022). Many microorganisms that are no longer biologically active can still be detected *via* high-throughput sequencing; therefore, it is important to understand the fermentation process by rapid analysis and sorting of the flora using flow cytometry combined with the absolute characterization of the microorganisms using high-throughput sequencing.

As an important functional community on stacked fermented, bacteria can produce various enzymes and plentiful Baijiu aroma substances or their precursors, which give Baijiu a unique flavor. *Lactobacillus*, *Weissella*, *Pediococcus*, *Staphylococcus*, *Thermoactinomyces*, and *Bacillaceae* were detected at the beginning of the stacking process. These dominant strains have also been detected as the dominant microorganisms in Daqu (Fan et al., 2019; Shang et al., 2022). *Lactobacillus*, which shows relatively high abundance in 1–3 rounds of stacking, is a common microorganism in the fermentation process of soy sauce white wine, which can further produce esters with organic acids and alcohols (Cappello et al., 2017) and inhibit the growth of spoilage bacteria by secreting bacteriocins to maintain an acidic brewing environment (Li et al., 2020). *Acetobacter* can oxidize glucose or ethanol to acetic acid, which in turn produces esters that are thought to enhance the fruit flavors in Baijiu. It has also been detected as a dominant strain in the fermentation process of rice-flavored Baijiu and the surface layer of high-temperature Daqu (Chen et al., 2020; Hu et al., 2021). *Weissella*, which ferments glucose to produce ethanol and acetic acid, was the dominant genus in all three rounds of the initial accumulation phase. It is widely present in fermented food, milk, and vegetables. (Fusco et al., 2015; Cheaito et al., 2020), and is generally considered an important functional genus in Baijiu fermentation, having been detected in fermented grains and Daqu (Wang P. et al., 2017; Wang X. et al., 2017). After cell sorting, we identified bacteria with activity in the samples that were not identical to the results of high-throughput sequencing. The main active bacteria are *Chitinophaga*, which can synthesize active substances (Huq and Akter, 2021), but there is very little research in sauce-flavor Baijiu. Bacteria from the genera *Herbaspirillum* can promote the growth of Sorghum (Kuramae et al., 2020), have also been detected during solid-state fermentation of Chinese baijiu, but have not attracted the attention of researchers due to low levels and lack of in-depth studies.

The sequencing results of the fungus revealed that *Issatchenkia* was consistently highly abundant (15.23–98.21%) in all three rounds. *Issatchenkia* spp. have good heat and alcohol tolerance and are efficient ethanol and ester producers (Dhaliwal et al., 2011; Seong et al., 2017). In addition, various aroma- and ester-producing yeasts were detected in these samples. Flow cytometric sorting of live bacteria at the end of the third round of stacking showed a very high abundance of *Aspergillus*, which secretes a large amount of protease during fermentation, catalyzing the hydrolysis of proteins and peptides and contributing to the production of a large amount of sauce-flavor precursors it also plays an important role in fermentation and saccharification (Jin et al., 2017). *Pichia* is a heat-tolerant yeast, and one study noted that abundant *Pichia* and *Candida* were detected in sauce-flavor Baijiu production tools on indoor floors and the hands of workers (Zhang et al., 2021). It remained active after high-temperature stacking was completed, allowing high production of alcohols, organic acids, and esters (Jiang et al., 2019). In addition, other active aromatic and ester-producing yeasts, such as *Candida*, *Italomyces*, *Papiliotrema*, and *Wickerhamomyces*, were detected at the end of the stack, all of which provided a power base for fermentation in the tank.

High-throughput sequencing dose not distinguish dead cells, which limited the understanding of microbial community structure, and after flow cytometry and cell sorting our collection of active microorganisms was achieved. Moreover, flow cytometry is a rapid with a low detection limit (Kupschus et al., 2022), our tests took only a few hours from sample preparation to completion of the cytometric analysis. Thus, this method is an excellent tool for dynamic monitoring. Typical flow cytometry applications have enabled the analysis of microbial communities in water environments and the detection of cell density in drinking water (Favere et al., 2020). In more complex matrices, flow cytometry has successfully monitored changes in up to 15 subpopulations of sludge (Gunther et al., 2012). Our study illustrates the potential of flow cytometry to detect microorganisms in complex environments, such as Chinese Baijiu. van de Velde et al. (2022) studied mixtures from intestinal flora and found that trends in 16SrRNA results were well predicted by flow cytometry, and the study also reported the efficiency of labeling *E. coli* with fluorescent probes to differentiate between species. This study indicated that designing specific probes for the corresponding species could facilitate the retrieval of species information in complex matrices accurately and rapidly using flow cytometry.

In summary, we used flow cytometry combined with high-throughput sequencing to investigate microbial flora in complex matrices. This method allowed for the real-time monitoring of the activity and changes in the abundance of bacteria, yeast, and mold during the brewing of sauce-flavored Baijiu. The findings suggest the potential of the investigated method to overcome the limited microbial discrimination capability of current flow cytometry methods. Moreover, by developing real-time flow cytometry and fluorescent probes, flow cytometry can be integrated into various fields to address the complex and changing microbial environment.

## Data availability statement

The datasets presented in this study can be found in online repositories. The names of the repository/repositories and accession number(s) can be found below: https://www.ncbi.nlm.nih.gov/, PRJNA944588.

## Author contributions

JW and RW designed the experiments. ZZ performed the experiments. YW, ZP, and PD analyzed the results. XD and GZ contributed to the reagents and materials. CW and PL drafted the manuscript. All authors read and approved the final manuscript.
