# Supplementary material for: Exploration of microbiome diversity of stacked fermented grains by flow cytometry and cell sorting
Source: Front Microbiol. 2023 Mar 27;14:1160552. doi: 10.3389/fmicb.2023.1160552 (PMC10083240; doi:10.3389/fmicb.2023.1160552)
Supplement: Supplementary file 1 [file Data_Sheet_1.pdf]

Supplementary Material

Exploration of microbiome diversity of stacked fermented grains by flow cytometry and cell sorting

Ziyang Zhang , Yanwei Wei, Zehao Peng, Peng Du, Xinyong Du, Guoying Zuo, Chaoqing Wang, Piwu Li, Junqing Wang, Ruiming Wang

*** Correspondence:**

Junqing Wang, and Ruiming Wang

E-mail: wjqtt.6082@163.com (Junqing Wang), and ruiming3k@163.com (Ruiming Wang)

1 Supplementary Figures

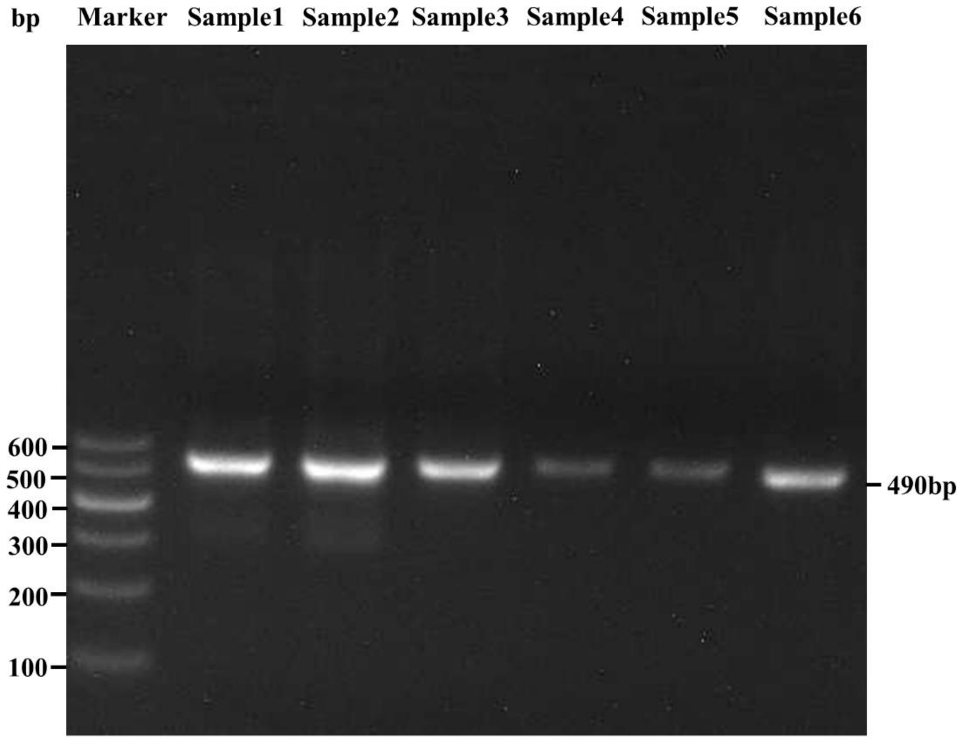

Supplementary Figure 1. Bacterial amplification gel image of stacked fermented grains, the sample order from left to right are DJ1, DJ1RJ, DJ2, DJ2RI, DJ3, DJ3RJ.

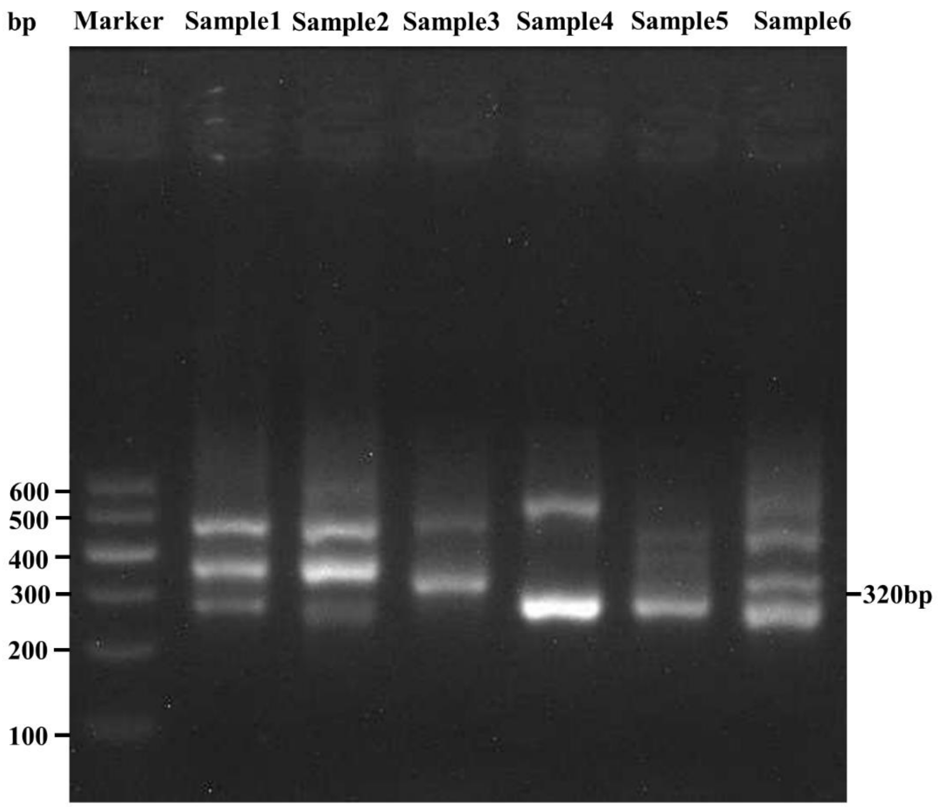

Supplementary Figure 2. Fungal amplification gel image of stacked fermented grains, the sample order from left to right are DJ1, DJ1RJ, DJ2, DJ2RI, DJ3, DJ3RJ.

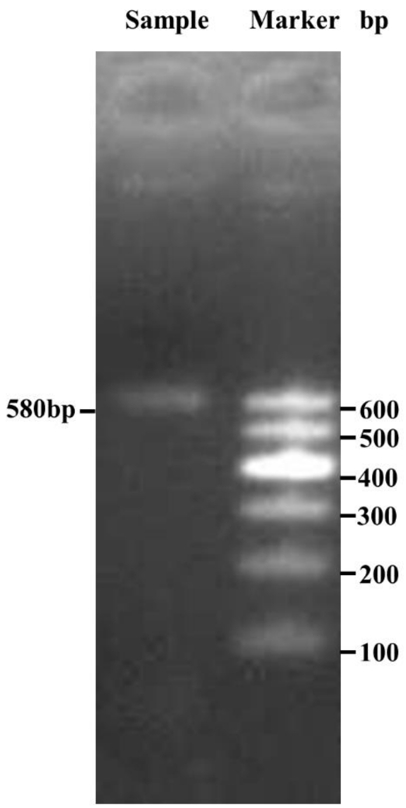

Supplementary Figure 3. Image of bacterial amplification gel after flow cytometric sorting.

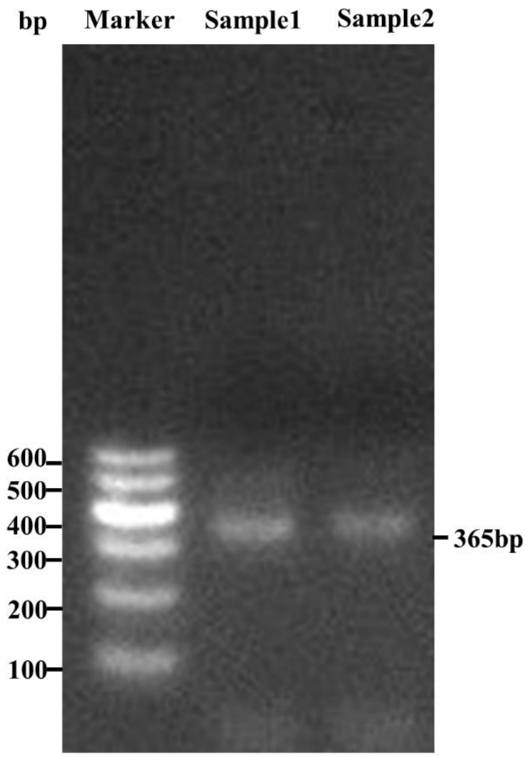

Supplementary Figure 4. Image of fungal amplification gel after flow cytometric sorting, The sample order from left to right are mould, yeast.
